# Supplementary material for: In Silico Identification and Validation of Pyroptosis-Related Genes in Chlamydia Respiratory Infection
Source: Int J Mol Sci. 2023 Sep 1;24(17):13570. doi: 10.3390/ijms241713570 (PMC10488104; doi:10.3390/ijms241713570)
Supplement: Supplementary file 1 [file ijms-24-13570-s001.zip › ijms-2569718-supplementary.pdf]

Supplementary Table S1. Sequences of primers used in this study.

Gene name	Forward primer (5’→3’)	Reverse primer (5’→3’)
Tnfα	CTGAACTTCGGGGTGATCGG	GGCTTGTCACTCGAATTTTGAGA
IL1β	GAAATGCCACCTTTTGACAGTG	ATCTTTTGGGGTCCGTCAACT
Tlr2	CACCACTGCCCGTAGATGAAG	AGGGTACAGTCGTCGAACTCT
Tlr9	ATGGTTCTCCGTCGAAGGACT	GAGGCTTCAGCTCACAGGG
Nlrp3	ATTACCCGCCCGAGAAAGG	TCGCAGCAAAGATCCACACAG
Zbp1	AAGAGTCCCCTGCGATTATTTG	TCTGGATGGCGTTTGAATTGG
Mefv	TCATCTGCTAAACACCCTGGA	GGGATCTTAGAGTGGCCCTTC
Tnfaip3	GAACAGCGATCAGGCCAGG	GGACAGTTGGGTGTCTCACATT
